# Reduced Body Fat and Epididymal Adipose *Apelin* Expression Associated With Raspberry Ketone [4-(4-Hydroxyphenyl)-2-Butanone] Weight Gain Prevention in High-Fat-Diet Fed Mice

**DOI:** 10.3389/fphys.2021.771816

**Published:** 2021-11-23

**Authors:** Lihong Hao, Nicholas T. Bello

**Affiliations:** Department of Animal Sciences, School of Environmental and Biological Sciences, Rutgers, The State University of New Jersey, New Brunswick, NJ, United States

**Keywords:** frambinone, *p*-hydroxybenzyl acetone, rasketone, *α*-glucosidase inhibitor, appetite suppressant, weight supplement

## Abstract

Raspberry ketone [4-(4-hydroxyphenyl)-2-butanone] is a natural aromatic compound found in raspberries and other fruits. Raspberry ketone (RK) is synthetically produced for use as a commercial flavoring agent. In the United States and other markets, it is sold as a dietary supplement for weight control. The potential of RK to reduce or prevent excessive weight gain is unclear and could be a convergence of several different actions. This study sought to determine whether acute RK can immediately delay carbohydrate hyperglycemia and reduce gastrointestinal emptying. In addition, we explored the metabolic signature of chronic RK to prevent or remedy the metabolic effects of diet-induced weight gain. In high-fat diet (HFD; 45% fat)-fed male mice, acute oral gavage of RK (200 mg/kg) reduced hyperglycemia from oral sucrose load (4 g/kg) at 15 min. In HFD-fed female mice, acute oral RK resulted in an increase in blood glucose at 30 min. Chronic daily oral gavage of RK (200 mg/kg) commencing with HFD access (HFD_RK) for 11 weeks resulted in less body weight gain and reduced fat mass compared with vehicle treated (HFD_Veh) and chronic RK starting 4 weeks after HFD access (HFD_RKw4) groups. Compared with a control group fed a low-fat diet (LFD; 10% fat) and dosed with vehicle (LFD_Veh), glucose AUC of an oral glucose tolerance test was increased with HFD_Veh, but not in HFD_RK or HFD_RKw4. Apelin (*Apln)* gene expression in epididymal white adipose tissue was increased in HFD_Veh, but reduced to LFD_Veh levels in the HFD_RK group. Peroxisome proliferator activated receptor alpha (*Ppara)* gene expression was increased in the hepatic tissue of HFD_RK and HFD_RKw4 groups. Overall, our findings suggest that long term daily use of RK prevents diet-induced weight gain, normalizes high-fat diet-induced adipose *Apln*, and increases hepatic *Ppara* expression.

## Introduction

Raspberry ketone {RK; [4-(4-hydroxyphenyl)-2-butanone]} is a natural phenolic compound derived from red raspberries (Borejsza-Wysocki et al., 1992; Borejsza-Wysocki and Hrazdina, 1994). It is the primary flavor and aroma compound of raspberries and considered safe for food and cosmetic applications in small quantities (Bredsdorff et al., 2015; Administration, 2019). Similar to other naturally derived products with functional hydroxyl groups, such as capsaicin, p-synephrine, and ephedrine, RK has also been investigated for its potential anti-obesity effects (Morimoto et al., 2005). Several *in vitro* studies have shown that RK has effects on fat metabolism by suppressing pre-adipocyte differentiation and fat accumulation, increasing lipolysis, and increasing fat oxidation (Park, 2010, 2015; Leu et al., 2017, 2018; Tsai et al., 2017). Previous studies have demonstrated that oral RK administered coincidently with access to a high-fat diet (HFD) for 2–10 weeks prevents diet-induced weight gain and adiposity in mice (Morimoto et al., 2005; Kshatriya et al., 2019, 2020). The notion that RK acts directly on white adipose tissue is supported by its rapid absorption (Tmax ∼ 15 min) and distribution into white adipose tissue following an acute oral gavage of RK (200 mg/kg) (Zhao D. et al., 2020). Another possible weight control mechanism for RK could be from actions on *α*-glucosidase. *α*-Glucosidase is an essential intestinal enzyme for carbohydrate digestion, and it functions to hydrolyze carbohydrates. Pre-clinical and clinical studies have shown that *α*-glucosidase inhibitors, such as acarbose, have antidiabetic effects by inhibiting carbohydrate hydrolysis and reducing postprandial blood glucose levels (Martin and Montgomery, 1996). *In vitro* and *in silico* enzyme kinetics studies have indicated that RK has rapid and reversible *α*-glucosidase inhibition, and that the hydroxyphenyl ring of RK potentially interacts with key residues on active sites of the enzyme (Xiong et al., 2018). Related to actions on postprandial glycemia and similar to other naturally derived phenolic compounds (Li et al., 2020; Zhao X. et al., 2020), RK could have additional actions on gastrointestinal function. Indeed, orally administered RK has been demonstrated to have gastroprotective effects on the development of ethanol-induced gastritis (Badr et al., 2019). Taken together, the potential effects of RK could be from several integrative actions to promote body weight control.

Despite the consumer popularity of RK as a dietary supplement for weight loss, a clear mechanism of action for weight loss has not been characterized. Previously, we have demonstrated that daily oral gavage of RK *prevented* HFD-induced weight gain after 4-week coadministration of HFD and RK (Kshatriya et al., 2019). For this study, we examined whether the chronic oral gavage administration of RK has the potential to *reduce* 4-week HFD-induced weight gain. To determine the weight gain prevention potential, we investigated the expression of genes related to inflammation, metabolism, and body weight homeostasis to determine the metabolic signature of RK for management of HFD-induced weight gain. In addition, we examined whether acute RK has actions to delay gastric emptying and acts to reduce carbohydrate meal-induced hyperglycemia to further characterize RK potential as a weight gain-preventative agent (Xiong et al., 2018).

## Materials and Methods

### Mice

C57BL/6J mice were purchased from The Jackson Laboratory (Bar Harbor, ME, United States). All the mice were housed, four mice per cage, upon arrival and were acclimated on standard chow (Purina Mouse Diet 5015, 25.34% fat, 19.81% protein, 54.86% CHO, 3.7 kcal/g; Lab Diet, St. Louis, MO, United States) for one week. They had free access to water, unless otherwise noted, and maintained on a 12-h light and 12-h dark cycle with lights on from 0500 to 1700 h. Oral gavage was performed using single-use, sterile plastic feeding tubes (20 ga × 30 mm; cat# FTP-20-30, Instech Laboratories, Plymouth Meeting, PA, United States). The animal care protocol was approved by the Institutional Animal Care and Use Committee of Rutgers University (OLAW #A3262-01); Protocol #999900014.

### Oral Carbohydrate Meal Test

A separate set of male and female C57BL/6J mice (*n* = 24 per sex, 4 weeks old) was fed a high-fat diet (HFD; D12451; Research Diets, Inc., New Brunswick, NJ United States; 45% fat, 20% protein, 35% carbohydrate; 4.73 kcal/g), and six male and six female mice were fed with sucrose matched low-fat diet (LFD; D12450H; Research Diets, Inc., New Brunswick, NJ United States; 10% fat, 20% protein, 70% carbohydrate; 3.85 kcal/g). The mice were fed for a period of 17 weeks and subjected to carbohydrate meal tests in the following 2 weeks with a 7-day washout between treatments. This long exposure to the HFD was utilized as a procedure to produce an alteration in blood dysregulation to facilitate the examination of *in vivo α*-glucosidase activity. A similar approach has been used in the examination of other natural products (Zhang et al., 2012). In addition, acarbose was administered as a positive control for *α*-glucosidase inhibition (Zhang et al., 2012). All the mice were food-deprived overnight (18 h), and baseline blood glucose levels were obtained from a tail nick. Half of the HFD fed mice (*n* = 12) were orally gavaged with acarbose (Acar, 5 mg/kg; cat# A0802; ≥ 98%; LKT Labs, St. Paul, MN, United States), and the other half were orally gavaged with raspberry ketone [RK, 200 mg/kg; 4(4-hydroxyphenyl)-2-butanone; 99%; cat# 178519; Lot# MKBQ9010V; Sigma Aldrich, St. Louis, MO, United States]. A reference sample of RK from the lot number was deposited in a secure, climate-controlled repository (Yuan et al., 2019). The LFD-fed mice were given vehicle (Veh; 50% propylene glycol, 40% water, and 10% dimethyl sulfoxide (DMSO) as a vehicle control immediately after the baseline blood glucose test. Sixty minutes later, all the mice were given oral gavage of sucrose (4 g/kg) or starch (3 g/kg). Blood samples were collected for glucose levels at −60, 0, 15, 30, 60, and 120 min.

### Gastric Emptying and Small Intestine Transit Measurement With Phenol Red Method

Gastric emptying and small intestine transit rate were measured according to the methods of Amira and colleagues (Amira et al., 2005). Briefly, a separate set of C57BL/6J mice (males: *n* = 10; females: *n* = 23) at age of 12 weeks was single housed and fed HFD for 2 weeks. The mice were fasted for 4 h but had free access to water. They were then orally gavaged a test meal containing phenol red (0.1%) and RK (200 mg/kg, males: *n* = 5; females: *n* = 12) or vehicle (males: *n* = 5; females: *n* = 11). Three RK and four vehicle mice were euthanized immediately after dosing (0 min), and the other mice were euthanized after 30 min. The stomach and small intestine were dissected with pylorus and cardia clamped. The stomach was transferred and homogenized in 10 ml.1N NaOH. After sedimentation at room temperature for 60 min, 5 ml of supernatant was mixed with 0.5 ml 33% trichloroacetic acid to precipitate any proteins and centrifuged at 3,000 rpm for 30 min. The supernatant was transferred to a new tube, and an equal volume of 0.5N NaOH was added to intensify the phenol red color. Phenol red concentration was measured by absorbance at 560 nm using a spectrophotometer. GE rate was calculated as follows: (1-absorbance at 30 min/absorbance at 0 min) × 100.

The whole length of the small intestine was measured after it was dissected. The intestine was opened from the end part and a drop of 0.1 N NaOH was used to localize the end point that the test meal reached. The length between the end point of test meal and the starting part of the intestine was defined as the distance traveled by the test meal. Intestinal transit rate was calculated as the ratio of the distance traveled by the test meal to the total length of small intestine × 100.

### Raspberry Ketone Daily Chronic Dosing

Thirty-two male mice (C57BL/6J; 8-week-old) were equally separated into four groups by body weight, with *n* = 8 per group, and fed either HFD or sucrose-matched LFD. All the mice also simultaneously received a daily oral gavage of vehicle or 200 mg/kg RK for 12 weeks. The four diet-compound groups were as follows: LFD_Veh, HFD_Veh, HFD_RKw4, and HFD_RK. Mice in the HFD_RKw4 group were orally gavaged with vehicle for the first 4 weeks (28 days) and switched to 200 mg/kg RK for the following 8 weeks. Mice in the other three groups were on their initial diet and treatment for over 12 weeks (84 days). All the mice had free access to water and respective diet at all times, unless otherwise noted and maintained on a 12-h light and 12-h dark cycle with lights on from 05:00 to 17:00 h. The mice were weighed daily, and food consumption was measured every week throughout the entire dosing period. Daily dosing was performed between 08:30 and 10:00 h.

### Body Composition on Week 11

Mice lean, fat, and fluid mass were measured using an EchoMRI 3-in-1 (Echo Medical Systems, Houston, TX, United States) body composition analyzer. Body composition was assessed a day (Day 0) before the initiation of diet and daily oral treatment and on week 11 (Day 77).

### Insulin Tolerance Test on Week 9 and Oral Glucose Tolerance Test on Week 10

Insulin Tolerance Test (ITT) was performed on week 9. The mice were fasted for 5 h and injected intraperitoneally (IP) with 0.75 U/Kg Humulin R (Henry Schein, Melville, NY, United States). Blood glucose levels were measured at 0, 15, 30, 60, 90, 120, and 180 min. The mice were returned to home cages and given food and water immediately after the ITT. The mice were not administered RK or Veh during the day of ITT. Oral dosing of compounds was resumed a day after the ITT.

Oral glucose tolerance test was performed on week 10. The mice were fasted for 6 h and given a dose of glucose (2 g/kg) orally. Blood glucose levels were measured at 0, 15, 30, 60, 90, 120, and 180 min. The mice were returned to home cages and given food and water immediately after the Oral Glucose Tolerance Test (OGTT) but were not given RK or Veh. Oral dosing of compounds was resumed a day after the OGTT (Supplementary Figure).

### Blood and Tissue Collection and Analysis

All the mice were food-deprived in new clean cages for 5 h (Day84). The mice were then deeply anesthetized under isoflurane mixed with oxygen and exsanguinated followed by decapitation. Blood was collected in EDTA tubes by cardiac puncture under deep isoflurane anesthesia, and 50-ul whole blood was analyzed by Element HT5 Hematology Analyzer (Heska Corporation, Loveland, CO, United States). The remaining plasma was used for plasma biomarkers analysis.

Brown adipose tissue and white adipose tissue including inguinal and epididymal adipose tissue, liver and mediobasal hypothalamus (2 mm dorsoventral block; 0.02 mm Bregma to −2.54 mm Bregma) were collected in RNase later and stored at −80°C.

#### Plasma Biomarker Assay

Plasma triglyceride, free fatty acids, cholesterol, and insulin were analyzed using a commercially available triglyceride quantification kit (Abcam, Cambridge, MA, United States), free fatty acids quantification kit (Millipore Sigma, St. Louis, MO, United States), HDL and LDL/VLDL cholesterol quantification kit (MyBioSource, San Diego, CA, United States), and mouse insulin enzyme-linked immunosorbent assay (ELISA) kit (Millipore Sigma, St. Louis, MO, United States).

#### RNA Extraction, and Reverse Transcription and Quantitative Real-Time Polymerase Chain Reaction for Adipose Tissue

Adipose tissue RNA was extracted using a standard TRIzol extraction combined with Machery-Nagel Nucleospin RNA extraction kit. DNase I was used to minimize any genomic DNA contamination. RNA quantity was determined using a NanoDrop ND-2000 spectrophotometer (Thermo Fisher Scientific Inc., Waltham, MA, United States).

Complementary DNA was synthesized from 200 ng of total RNA in a 20-ul reaction system containing a Superscript III (Thermo Fisher Scientific, Inc., Waltham, MA, United States) reverse transcriptase, 4 μl 5 × SS buffer, 1.25 μl 100 mM dNTP, 100 ng random hex primers (Promega Corp, Madison, WI, United States), 40 U/μl Rnasin (Promega Corp, Madison, WI, United States), and 100 mM dithiothreitol in diethylpyrocarbonate-treated water. Reverse transcription was conducted using the following protocol: 25°C for 5 min, 50°C for 60 min, 70°C for 15 min and then cooling to 4°C for 5 min. The cDNA was diluted 1:20 using nuclease free water and stored at −20°C. Naive tissue RNA was used for positive and negative controls (no reverse transcriptase) and processed simultaneously with the experimental samples.

For quantitative real-time polymerase chain reaction (PCR), a CFX opus 96 real-time PCR instrument (Bio Rad Laboratories, Inc, Hercules, CA, United States) was used for gene amplification from 4 μl of cDNA using SsoAdvancedTm Universal SYBR^®^ Green Supermix (Bio Rad Laboratories, Inc, Hercules, CA, United States), primers, and nuclease free water. The amplification protocols for all genes are as follows: initial denaturation at 95°C for 3 min, followed by 40 cycles of denaturation at 95°C for 10 s, annealing at 60°C for 45 s, and completed with a dissociation step for melting point analysis with 60 cycles of 95°C for 10 s, 65 to 95°C (in increments of 0.5°C) for 5 s, and 95°C for 5 s.

Relative mRNA expression was calculated using the δδ cycle threshold (CT) method. The CT for each transcript was determined by consistently setting the baseline threshold at the lowest point of the exponential curve where the slope of the curve was steepest for all plates. Geometric means of the reference genes Gapdh and Hprt were used to calculate δCT. The relative linear quantity of target molecules was calculated using the formula 2^δδCT. All gene expression data were expressed as an n-fold difference relative to the LFD_Veh group.

Primers for the following genes were purchased from BioRad Laboratories: adrenoceptor alpha-1A (*Adra1a*), adrenoceptor alpha-1B (*Adra1b*), adrenoceptor alpha-2B (*Adra2b*), adrenoceptor alpha-2C (*Adra2c*), arachidonate 15-lipoxygenase (*Alox15*), apelin (*Apln*), apelin receptor (*Aplnr*), beta-3 adrenergic receptor (*b3AR*), gamma-aminobutyric acid receptor subunit gamma-2 (*Gabrg2*), glutamate ionotropic receptor AMPA-type subunit 1 (*Gria1*), heme oxygenase (*Hmox1*), interleukin 6 (*Il-6*), hormone-sensitive lipase (*Lipe*), lipoprotein lipase (*Lpl*), neuropeptide Y(*Npy*), proopiomelanocortin (*Pomc*), peroxisome proliferator-activated receptor alpha (*Ppara*), peroxisome proliferator-activated receptor gamma (*Pparg*), peroxisome proliferator-activated receptor-gamma coactivator (PGC)-1alpha (*Pparg1ca*), tumor necrosis factor(*TNF*), uncoupled protein 1 *(Ucp1)*, and uncoupled protein 2 *(Ucp2)*. Primers for housekeeping genes *Gapdh and Hprt* were synthesized by Life Technologies, Inc. (Life Technologies, Inc., Carlsbad, CA, United States), and primer sequences were as follows:

*Gapdh:*
Forward: TGACGTGCCGCCTGGAGAAA; reverse: AGTG TAGCCCAAGATGCCCTTCAG.
*Hprt*:
Forward: GCTTGCTGGTGAAAAGGACCTCTCGAAG;
Reverse: CCCTGAAGTACTCATTATAGTCAAGGGCAT.

### Statistical Analyses

For daily body weight gain, weekly food intake, glucose response to insulin, or oral glucose administration, an analysis of variance (ANOVA) with repeated measures was performed. For the other measurements, one-way ANOVA was performed. When justified, Newman Keuls *post hoc* tests were performed. All the statistical analyses were performed using the Statistica 7.1 software (StatSoft; Dell Inc, Round Rock, TX, United States). All the data were represented as mean ± standard error of the mean (SEM), and a *p* value less than 0.05 was considered statistically significant.

## Results

### Raspberry Ketone Lowers Peak Glucose Levels in Response to Oral Sucrose Load in Male Obese Mice

**FIGURE 1:**
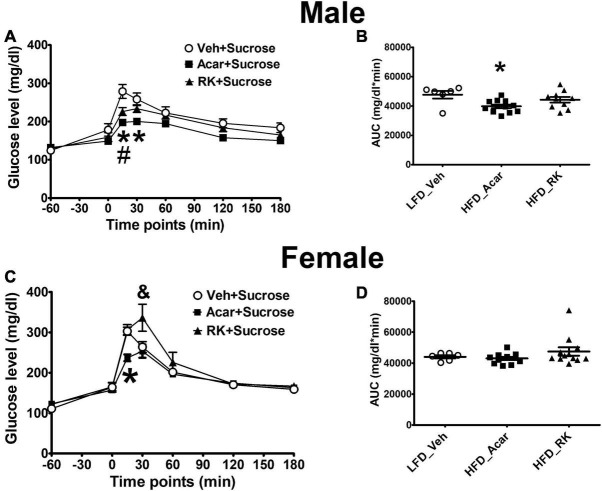
Acute effect of raspberry ketone (RK, 200 mg/kg) on glucose response to oral load of sucrose (4 g/kg) in male and female mice. Mice were fed with high-fat diet (HFD, 45% fat) or low-fat diet (LFD, 10% fat) for 17 weeks. RK, acarbose, and vehicle were administered 1 h prior to carbohydrate meal test. RK prevented peak glucose elevation **(A)** but not area under curve (AUC) of glucose **(B)** in male mice. RK increased blood glucose at 30 min **(C)** but not glucose AUC in female mice **(D)**. Data are represented as mean ± standard error of the mean (SEM). *p < 0.05 acarbose vs. vehicle, ^#^*p* < 0.05 RK vs. vehicle, and & *p* < 0.05 RK vs. vehicle and acarbose. Repeated measures with analysis of variance (ANOVA) for glucose response analysis; one way ANOVA for AUC analysis, followed by Newman Keuls *post hoc* testing. LFD_Vehicle (*n* = 6/sex); HFD_Acarbose (*n* = 12/sex); HFD_RK (males: *n* = 10; females: *n* = 12).

The HFD induced weight gain in the mice that were used to investigate the acute effect of RK on blood glucose response to sucrose or starch oral administration. The LFD-fed mice were used as controls. All the mice were on assigned diets for 17 weeks. The HFD-fed mice had significant increased body weight in both the male (44.7 ± 1 g) and female (32.1 ± 1.1 g) mice compared with the controls (males: 37 ± 1.4 g, *p* < 0.001; females: 22.2 ± 0.6 g, *p* < 0.001). Fasting glycemia in the LFD control male and female mice was 124 ± 5.2 and 111 ± 2.9 mg/dl, respectively. Fasting glucose levels were not significantly elevated in either the male (132 ± 4.2 mg/dl) or female (122 ± 2.4 mg/dl) HFD mice. For oral sucrose dosing in males, there were group [*F*(2, 25) = 5.9, *p* < 0.05], time [*F*(6, 150) = 110.7, *p* < 0.0005], and group X time [*F*(12, 150) = 4.6, *p* < 0.0005] effects. *Post hoc* testing revealed blood glucose levels were overall lower in acarbose (5 mg/kg) than vehicle group (*p* < 0.005). Blood glucose levels in the Acar group were significantly lower than in the vehicle group at 15 and 30 min (*p* < 0.05 for both), whereas blood glucose levels in the RK group was only significantly lower than in the vehicle-treated group at 15 min (*p* < 0.05) (Figure 1A). There was also an effect for AUC [*F*(2, 25) = 4.7, *p* < 0.05] with Acar having lower AUC than vehicle (*p* < 0.05) (Figure 1B). For oral sucrose dosing in females, there was a time [*F*(6, 162) = 84.5, *p* < 0.0005] and group X time [*F*(12, 162) = 3.5, *p* < 0.005]. *Post hoc* testing revealed that blood glucose in Acar was lower than in vehicle at 15 min (*p* < 0.05), and that RK dosing elevated blood glucose at 30 min compared with Acar and vehicle (*p* < 0.05 for both) (Figure 1C). There were no differences in AUC (Figure 1D). For oral starch, there were no group differences or group X time differences in male and females, (not shown). Notably, there were no differences in blood glucose levels at time 0, which was 60 min after the administration of RK, Acar, and vehicle in males and females in any of the carbohydrate meal tests.

### Raspberry Ketone Has No Effect on Gastric Emptying and Intestinal Transit

**FIGURE 2:**
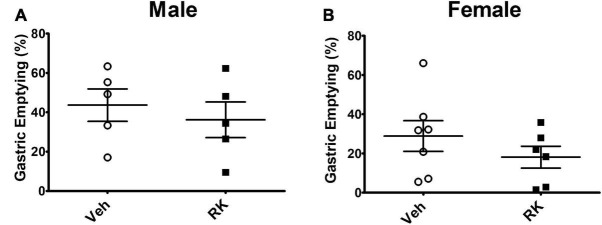
RK (200 mg/kg) does not accelerate the % emptying of phenol red from stomach in **(A)** male or **(B)** female mice compared with vehicle control. Data are represented as mean ± SEM. Vehicle (males: *n* = 5; females: *n* = 7); RK (males: *n* = 5; females: *n* = 6).

Gastric emptying rate was measured using a test meal containing phenol red. In the Veh group, the gastric emptying (GE) rate of test meal from stomach is around 43 and 30% for the male and female mice, respectively. RK did not significantly accelerate or delay test meal emptying in either male or female mice (Figure 2). Intestinal transit rate was measured and calculated in the female mice. The addition of RK to the test meal did not significantly increase intestinal transit (14.6 ± 5.6%) compared with the control mice (21.7 ± 9.3%).

### Daily Dosing of Raspberry Ketone Prevents High-Fat Diet-Induced Body Weight Gain, Reduces Fat Content, but Does Not Decrease Energy Intake From High-Fat Diet

**FIGURE 3:**
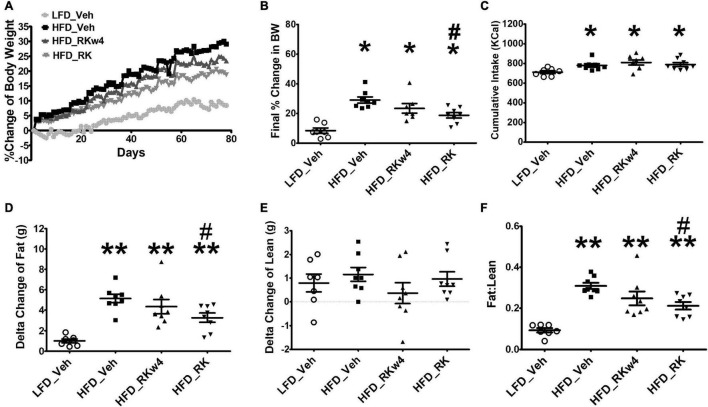
Daily RK (200 mg/kg) dosing prevented body weight gain in male mice. Groups were mice that were fed with a low-fat diet daily oral gavage with vehicle (LFD_Veh), high-fat diet oral gavage with vehicle (HFD_Veh), oral RK dosing initiated at 4 weeks of HFD (HFD_RKw4), and HFD and oral gavage RK initiated at the same time (HFD_RK). **(A)** Percent change of body weight from baseline over 78 days of diet intervention and daily dosing. **(B)** Percent change of body weight at Day 78. **(C)** Cumulative Kcal intake over 78 days. **(D)** Difference from Day 0 to Day 79 in fat mass and **(E)** Lean mass measured by EchoMRI, and **(F)** Fat: lean ratio. Data are represented as mean ± SEM. *p < 0.05 vs. LFD_Veh; **p < 0.0001 vs. LFD_Veh, ^#^*p* < 0.05 vs. HFD_Veh. Data were analyzed by one-way ANOVA followed by Newman Keuls *post hoc* testing (*n* = 7 for LFD_Veh; *n* = 8 per HFD group).

To determine whether RK has effects on reducing body weight or adiposity, all the mice were exposed to assigned diet and oral gavage treatment for 12 weeks. Daily bodyweight was recorded, and body composition was measured at the end of the study (after week 11, on day 78). The baseline body weights were 24.34 ± 0.32, 24.42 ± 0.49, 25.68 ± 0.52, and 25.03 ± 0.56 g for LFD_Veh, HFD_Veh, HFD_RKw4, and HFD_RK, respectively. Over the 11 weeks, there were treatment effect [*F*(3, 26) = 15.2, *p* < 0.0005], days effect [*F*(76, 1,976) = 189.3, *p* < 0.0005], and treatment X day effect [*F*(228, 1,976) = 4.8, *p* < 0.005]. *Post hoc* testing revealed that all the groups had increased body weight gain compared with LFD_Veh (*p* < 0.005). HFD_RK had reduced body weight gain compared with HFD_Veh (*p* < 0.05). On Day 25, HFD_RK had less body weight gain compared with the HFD_Veh group (*p* < 0.05). The difference between HFD_Veh and HFD_RK remained until the end of the study (*p* < 0.05 for all days). Because RK (200 mg/kg) dosing began on day 28 for the HFD_RKw4 group, an additional analysis of body weight gain from day 29 to the end of the study was performed (last 8 weeks). There were treatment [*F*(3, 26) = 14.4, *p* < 0.0005], time [*F*(49, 1,274) = 93.2, *p* < 0.005], and group x time [*F*(147, 1,274) = 3.2, *p* < 0.005] effects. *Post hoc* testing indicated that there was a difference between HFD_Veh and HFD_RK (*p* < 0.05), but that there were no group differences between HFD_Veh and HFD_RKw4 and that there were no group differences between HFD_RK and HFD RKw4 (Figure 3A). There was an overall significant effect of final % body weight change on Day 78 [*F*(3, 26) = 14.5, *p* < 0.0005]. *Post hoc* testing indicated that HFD_Veh, HFD_RKwk4, and HFD_RK had increased body weight gain compared with LFD_Veh (*p* < 0.005 for all). There was significantly less body weight gain in HFD_RK compared with HFD_Veh (*p* < 0.005; Figure 3B).

For cumulative caloric intake, there was an overall difference [*F*(3, 27) = 4.1, *p* < 0.05]. All mice on the HFD group had higher cumulative energy intake compared with the LFD-fed mice (*p* < 0.05, Figure 3C). There were no differences among the HFD_Veh, HFD_RKw4, and HFD_RK groups.

The assessment of body composition occurred on Day 0 and Day 77. There was an overall significant effect for the change in fat mass [*F*(3, 27) = 12.8, *p* < 0.005]. *Post hoc* testing indicated that all the HFD-fed groups had increased fat mass compared with the LFD vehicle group (*p* < 0.005 for all). In addition, HFD_RK had lower fat mass gain than HFD_Veh (*p* < 0.05, Figure 3D). There were no differences in gain in lean mass among the groups (Figure 3E), but there was an effect when expressed as a fat/lean ratio [*F*(3, 27) = 16.2, *p* < 0.0005]. The differences in the ratio were similar to the group difference in fat mass, with all the HFD-fed groups having a higher ratio than LFD_Veh (*p* < 0.005), and HFD_RK had a lower ratio than HFD_Veh (*p* < 0.005 Figure 3F).

### Daily Dosing of Raspberry Ketone Normalizes Glucose Tolerance in High-Fat Diet-Fed Mice

**FIGURE 4:**
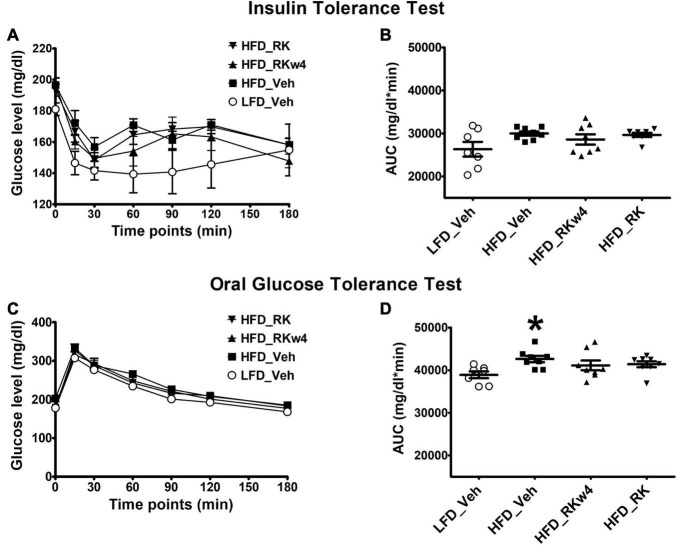
Insulin tolerance test (ITT) and glucose tolerance test (OGTT) on Weeks 9 and 10, respectively, of daily oral gavage of RK (200 mg/kg) or vehicle (Veh) in mice. Groups were mice that were fed a low-fat diet daily oral gavage with vehicle (LFD_Veh), high-fat diet oral gavage with vehicle (HFD_Veh), oral RK dosing initiated at 4 weeks of HFD (HFD_RKw4), and HFD and oral gavage RK administered (HFD_RK). RK had no effect on glucose response in the ITT **(A,B)**, but normalized responses in the OGTT **(C,D)**. Data are represented as mean ± SEM. *p < 0.05 vs. LFD_Veh. Repeated measures with ANOVA for glucose response analysis; one way ANOVA for AUC followed by Newman Keuls *post hoc* testing (*n* = 7 for LFD_Veh; *n* = 8 per HFD group).

We performed ITT and OGTT tests to determine whether RK improves blood glucose response to insulin or glucose administration due to its body weight and adiposity reduction effect. On Week 9, for ITT, there was a significant effect of treatment [*F*(3, 27) = 3.3, *p* < 0.05] with *post hoc* testing, indicating that LFD_Veh had the lowest blood glucose. There was also an effect for time [*F*(6, 162) = 19, *p* < 0.05] but not for treatment X time (Figure 4A). There were no significant effects of ITT on AUC [*F*(3, 27) = 2.3, *p* = 0.09] (Figure 4B). On week 10, for the OGTT, there was a treatment effect [*F*(3, 27) = 3, *p* < 0.05] with *post hoc* testing revealing lower overall blood glucose levels in LFD_Veh compared with HFD_Veh (*p* < 0.05). There was a time effect [*F* (6, 162) = 265.7, *p* < 0.005] but not treatment X time effect (Figure 4C). There was a significant effect of OGTT AUC [*F*(3, 27) = 3, *p* < 0.05] with only a difference between LFD_Veh and HFD_Veh (*p* < 0.05) (Figure 4D). There was a difference in baseline glucose for OGTT [*F*(3, 27] = 4.5, *p* < 0.05], with *post hoc* testing revealing HFD_Veh having the highest blood baseline blood glucose (*p* < 0.05 for all). The *post hoc* testing did not reveal differences among LFD_Veh, HFD_RK, and HFD_RKw4. Blood glucose values at OGTT were 177 ± 3.5 mg/dl for LFD_Veh, 202.5 ± 3.7 mg/dl for HFD_Veh, 184.1 ± 7.9 mg/dl for HFD_RKw4, and 178 ± 4.8 mg/dl for HFD_RK.

### Daily Dosing of Raspberry Ketone for 12 Weeks Alters Gene Expression in White Adipose and Hepatic Tissues

**FIGURE 5:**
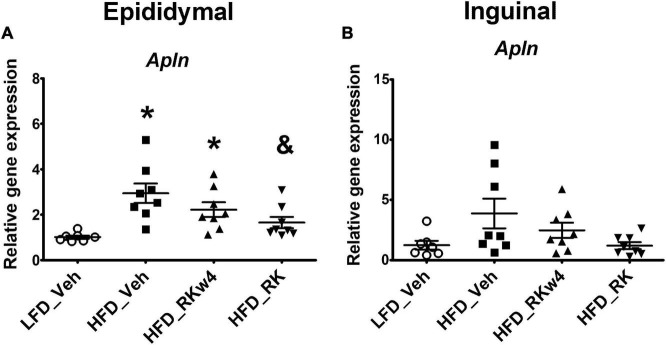
Relative gene expression for apelin (*Apln*) in epididymal **(A)** and inguinal **(B)** adipose tissue for mice with RK (200 mg/kg) or vehicle daily dosing for a period of 12 weeks. All gene expression data were expressed as an n-fold difference relative to the mean of the LFD group. Data are represented as mean ± SEM. *p < 0.05 vs. LFD_Veh and & *p* < 0.05 vs HFD_Veh; One-way ANOVA followed by Newman Keuls *post hoc* analysis (*n* = 7 for LFD_Veh; *n* = 8 per HFD group).

For gene expression in white adipose tissue, there was an overall significant effect of apelin (*Apln*) expression in epididymal adipose tissue [*F*(3, 27) = 6.9, *p* < 0.01]. *Post hoc* testing revealed that HFD_Veh and HFD_RKw4 had increased *Apln* expression compared with LFD_Veh (*p* < 0.005 and *p* < 0.05, respectively). However, HFD_RK was only different from HFD_Veh (*p* < 0.05) (Figure 5A). In inguinal adipose tissue, there was only a trend effect for *Apln* expression [*F*(3, 27) = 2.9, *p* = 0.056]. Raspberry ketone tends to suppress *Apln* expression by around 69% when compared with the HFD control group (Figure 5B). All the other genes that were examined in this study are listed in Table 1. There was an overall significant effect on peroxisome proliferator-activated receptor-gamma coactivator (PGC)-1alpha (*Ppargc1a*) expression in epididymal adipose tissue [*F* (3, 27) = 3, *p* < 0.05]. However, the *post hoc* analysis did not indicate statistical differences among the groups (Table 1). In the liver, there was an effect [*F*(3, 27) = 5.7, *p* < 0.005] for peroxisome proliferator-activated receptor alpha (*Ppara*) expression. The *post hoc* analysis revealed that HFD_RKw4 and HFD_RK had increased *Ppara* expression compared with LFD_Veh (*p* < 0.05 and *p* < 0.005, respectively). In addition, there was an effect [*F*(3, 27) = 3.7, *p* < 0.05] on *Ppara* expression in brown adipose tissue. The *post hoc* analysis showed that HFD vehicle had increased *Ppara* expression compared with LFD control (*p* < 0.05). *Ppara* expression in the RK-treated mice had no statistical difference when compared with LFD control or HFD control (Table 1).

**TABLE 1:** Gene expression in white adipose tissue, brown adipose tissue, liver, and brain.

	**LFD_Veh**	**HFD_Veh**	**HFD_RKw4**	**HFD_RK**	**P value**
**Epididymal**					
*Alox15*	1.184 ± 0.304	0.501 ± 0.118	0.950 ± 0.191	1.422 ± 0.462	0.176
*Apnr*	1.035 ± 0.120	1.039 ± 0.123	0.961 ± 0.076	1.297 ± 0.130	0.190
*Il-6*	1.055 ± 0.123	1.111 ± 0.178	1.173 ± 0.167	0.924 ± 0.171	0.731
*Lipe*	1.250 ± 0.374	0.785 ± 0.088	0.758 ± 0.079	1.611 ± 0.339	0.053
*Lpl*	1.343 ± 0.424	1.053 ± 0.291	0.722 ± 0.083	1.674 ± 0.410	0.210
*Pparg*	1.201 ± 0.286	0.821 ± 0.145	0.775 ± 0.065	1.141 ± 0.183	0.264
*Pparg1ca*	1.328 ± 0.416	0.464 ± 0.096	0.396 ± 0.074	1.112 ± 0.246	**0.048**
*TNF*	1.080 ± 0.188	1.127 ± 0.198	1.291 ± 0.227	1.111 ± 0.069	0.844
*Ucp2*	1.149 ± 0.249	1.161 ± 0.124	1.178 ± 0.127	1.818 ± 0.308	0.093
**Inguinal**					
*Alox15*	1.183 ± 0.277	0.877 ± 0.248	0.804 ± 0.238	1.055 ± 0.332	0.772
*Aplnr*	1.062 ± 0.150	2.392 ± 0.693	1.920 ± 0.323	1.405 ± 0.229	0.152
*Pparg*	1.030 ± 0.110	1.386 ± 0.247	1.531 ± 0.452	1.876 ± 0.382	0.385
*Tnf*	2.069 ± 0.110	1.991 ± 0.889	2.443 ± 1.316	1.433 ± 0.557	0.892
*Ucp1*	3.172 ± 1.724	0.102 ± 0.040	0.058 ± 0.033	2.128 ± 1.855	0.227
*Ucp2*	1.224 ± 0.273	2.200 ± 0.450	0.770 ± 0.157&	1.989 ± 0.397#	**0.021**
**Liver**					
*Hmox1*	1.031 ± 0.114	0.862 ± 0.041	0.910 ± 0.030	0.939 ± 0.070	0.390
*Lipe*	1.097 ± 0.181	1.319 ± 0.261	1.685 ± 0.218	1.829 ± 0.175	0.091
*Lpl*	1.056 ± 0.144	0.474 ± 0.052***	0.341 ± 0.033***	0.403 ± 0.057***	** < 0.0001**
*Ppara*	1.023 ± 0.085	1.231 ± 0.102	1.365 ± 0.067*	1.547 ± 0.102*	**0.004**
*Pparg*	1.057 ± 0.133	1.565 ± 0.136	1.352 ± 0.150	1.802 ± 0.288	0.069
*Pparg1ca*	1.026 ± 0.103	0.980 ± 0.146	0.924 ± 0.144	0.846 ± 0.096	0.772
**Mediobasal hypothalamus**					
*Adra1a*	1.026 ± 0.095	1.071 ± 0.048	1.152 ± 0.095	1.064 ± 0.162	0.871
*Adra1b*	1.014 ± 0.068	1.143 ± 0.066	1.157 ± 0.065	1.159 ± 0.096	0.508
*Adra2b*	1.378 ± 0.451	2.141 ± 0.384	2.385 ± 0.698	1.132 ± 0.355	0.240
*Adra2c*	1.016 ± 0.070	0.896 ± 0.086	0.942 ± 0.110	0.906 ± 0.090	0.804
*Apln*	1.011 ± 0.058	0.112 ± 0.073	1.020 ± 0.066	0.922 ± 0.083	0.322
*Aplnr*	1.139 ± 0.188	1.390 ± 0.304	1.082 ± 0.187	0.881 ± 0.127	0.385
*Gabrg2*	1.016 ± 0.076	1.153 ± 0.076	1.196 ± 0.043	1.085 ± 0.106	0.419
*Gria1*	1.016 ± 0.077	1.100 ± 0.104	1.027 ± 0.121	0.793 ± 0.119	0.228
*Npy*	1.007 ± 0.047	0.798 ± 0.092	0.761 ± 0.031	0.783 ± 0.082	0.078
*Pomc*	1.016 ± 0.138	1.130 ± 0.161	1.200 ± 0.273	1.387 ± 0.268	0.745
*Ppara*	1.019 ± 0.084	1.372 ± 0.135	1.349 ± 0.166	0.965 ± 0.166	0.112
*Ucp2*	1.005 ± 0.039	1.348 ± 0.271	1.348 ± 0.322	0.794 ± 0.140	0.253
**Brown adipose tissue**					
*b3AR*	1.024 ± 0.087	1.040 ± 0.145	1.005 ± 0.057	0.812 ± 0.059	0.295
*Ppara*	1.034 ± 0.113	2.094 ± 0338*	1.625 ± 0.177	1.562 ± 0.167	**0.024**
*Ucp1*	1.027 ± 0.097	1.415 ± 0.232	1.187 ± 0.059	1.199 ± 0.080	0.293
*Ucp2*	1.042 ± 0.130	1.311 ± 0.164	1.105 ± 0.104	1.033 ± 0.092	0.374

*Adrenoceptor alpha-1A *(Adra1a)*, adrenoceptor alpha-1B (*Adra1b*), adrenoceptor alpha-2B (*Adra2b*), adrenoceptor alpha-2C (*Adra2c*), arachidonate 15-lipoxygenase(*Alox15*), apelin(*Apln*), apelin receptor (*Aplnr*), beta-3 adrenergic receptor (*b3AR*), gamma-aminobutyric acid receptor subunit gamma-2 (*Gabrg2*), glutamate ionotropic receptor AMPA-type subunit 1(*Gria1*), heme oxygenase (*Hmox1*), interleukin 6 (*Il-6*), hormone-sensitive lipase (*Lipe*), lipoprotein lipase (*Lpl*), neuropeptide Y(*Npy*), proopiomelanocortin (*Pomc*), peroxisome proliferator activated receptor alpha (*Ppara*), peroxisome proliferator activated receptor gamma (*Pparg*), peroxisome proliferator-activated receptor-gamma coactivator (PGC)-1alpha (*Pparg1ca*), tumor necrosis factor(*TNF*), uncoupled protein 1 *(Ucp1)*, uncoupled protein 2 *(Ucp2).* All gene expression data were expressed as an n-fold difference relative to the mean of the LFD group. Data are represented as means ± standard error of the mean (SEM).*

*p < 0.05 vs. LFD_Veh and & *p* < 0.05 vs. HFD_Veh; ^#^*p* < 0.05 vs. HFD_RKw4; ***p < 0.0001 vs. LFD_Veh. One-way analysis of variance (ANOVA) followed by Newman Keuls *post hoc* testing (*n* = 7 for LFD_Veh; *n* = 8 per HFD group).

### Daily Dosing of Raspberry Ketone on Plasma Lipid Profile and Plasma Insulin and Hematological Profile

We also examined RK effect on adiposity metabolism-related plasma biomarkers and blood cells. The daily dosing of RK for 12 weeks had no significant effect on plasma triglyceride and free fatty acids levels (Table 2). HFD feeding increased total cholesterol concentrations compared with LFD control. RK dosing had no significant effect on total cholesterol levels (Table 2). There was an effect for insulin [*F*(3, 26) = 3.9, *p* < 0.05], and *post hoc* testing revealed that HFD_Veh had higher insulin levels than LFD_Veh and HFD_RKw4 (*p* < 0.05 for both) (Table 2). Whole blood hematology analysis did not reveal that RK dosing in a period of 12 weeks (HFD_RK) had any significant adverse effect. However, there was an overall significant effect on (LYM) lymphocytes [*F*(3, 27) = 4.2, *p* < 0.05] with *post hoc* testing revealing that HFD_RKw4 and HFD_RK had reduced LYM counts compared with LFD_Veh (*p* < 0.05 for both). The *post hoc* testing also indicated that HFD_RK had reduced LYM counts compared with HFD_Veh (*p* < 0.05) (Supplementary Table 1). In addition, there was an overall significant effect of percentage of neutrophils (NEU) [*F*(3, 27) = 3.4, *p* < 0.05]. *Post hoc* testing revealed an increase in NEU % in HFD_RKw4 compared with HFD_Veh (*p* < 0.05; Supplementary Table 1).

**TABLE 2:** Plasma lipid and insulin analysis for mice with RK or vehicle daily dosing for a period of 12 weeks.

	**LFD_Veh**	**HFD_Veh**	**HFD_RKw4**	**HFD_RK**
Total Cholesterol (ug/ul)	1.008 ± 0.036	1.364 ± 0.021***	1.389 ± 0.053***	1.340 ± 0.039***
HDL Cholesterol (ug/ul)	0.628 ± 0.024	0.729 ± 0.009**	0.729 ± 0.016**	0.698 ± 0.022
LDL/VLDL Cholesterol (ug/ul)	0.176 ± 0.023	0.274 ± 0.024	0.283 ± 0.035*	0.222 ± 0.021
HDL-to-LDL/VLDL ratio	4.034 ± 0.660	2.844 ± 0.355	2.852 ± 0.393	3.300 ± 0.302
Free fatty acid (um)	359.5 ± 30.61	292.0 ± 21.08	314.6 ± 15.43	309.1 ± 17.65
Triglyceride (ug/ul)	0.448 ± 0.040	0.441 ± 0.050	0.422 ± 0.046	0.398 ± 0.048
Insulin (ng/ml)	0.578 ± 0.141	1.039 ± 0.112*	0.616 ± 0.088&	0.731 ± 0.067

*High density lipoprotein (HDL); low density lipoprotein (LDL); very low density lipoprotein (VLDL). Data are represented as means ± standard error of the mean (SEM).*

*p < 0.05 vs. LFD_Veh; **p < 0.005 vs. LFD_Veh; ***p < 0.0001 vs. LFD_Veh; & *p* < 0.05 vs. HFD_Veh. One way ANOVA followed by Newman Keul’s *post hoc* testing.

*Cholesterol assay: *n* = 7 per group; Free fatty acid and Triglyceride assay: *n* = 7 for LFD_Veh; *n* = 8 per HFD group.*

## Discussion

Dietary supplements for weight loss are not validated for effectiveness and often overmarketed for their intended purposes. Several *in vitro* studies have demonstrated that RK affects lipid metabolism by reducing lipid accumulation and promoting lipolysis and fatty acid oxidation (Park, 2010, 2015; Leu et al., 2017, 2018; Tsai et al., 2017). This study was designed to evaluate the *in vivo* potential of RK for prevention and treatment of HFD-induced body weight gain. Based on our previous studies on C57Bl/6J mice (Kshatriya et al., 2019, 2020; Hao et al., 2020; Zhao D. et al., 2020), the oral dose of RK that achieves effective prevention of body weight gain without any evidence of toxicity is 200 mg/kg (Hao et al., 2020). To further identify the mechanism for weight gain prevention, the 200 mg/kg dose was used to determine the acute converging effects of weight gain prevention and the metabolic signature of long-term (>10 weeks) administration. Our results indicated that oral daily administration of RK (200 mg/kg) coincident with access to HFD prevents daily bodyweight gain, reduces adiposity, and alters the expression of genes related to adipocyte differentiation and lipolysis. To address the treatment potential of RK for excessive weight gain, we also investigated whether oral RK initiated 4 weeks after the start of high-fat diet access reduces accumulated body gain. Our major findings indicate that RK (200 mg/kg) was effective in preventing diet-induced body weight gain and adiposity, but not effective in reducing accumulated body weight gain in adult male mice. A generalized interpretation of our study is limited, because our long-term dosing experiments were performed in male mice only. Previous short-term (14 days) studies demonstrate reduced HFD-induced weight gain with oral RK (400 mg/kg) in males but not in females (Kshatriya et al., 2020). In addition, there was a dose-dependent sex difference of RK on meal patterns and sex-dependent difference in bioavailability following oral RK administration (Kshatriya et al., 2020; Zhao D. et al., 2020). Nonetheless, the sex-dependent effects of the long-term oral administration of RK on weight gain prevention and treatment remains to be determined.

Another major finding of this study was that RK had the potential to alter blood glucose level, which could be separate from the difference in body weight and adiposity levels. In the long-term study, after 10 weeks of HFD, we observed an increase in blood glucose AUC in response to an OGTT. Daily dosing with RK normalized the blood glucose AUC increased by HFD feeding. This suggests that RK potential to maintain normal glucose homeostasis is not secondary to the lower body weight in the RK-treated mice. A few possible mechanisms for RK to prevent diet-induced weight gain could be inhibition the digestion of carbohydrates or slowing of gastric emptying. An *in vitro* enzyme kinetic study suggested that RK can bind to *α*-glucosidase and acts as a rapid and reversible *α*-glucosidase inhibitor (α-GI) (Xiong et al., 2018). Several *α*-GIs, such as acarbose, have been used for blood glucose control (Martin and Montgomery, 1996). In a separate set of HFD-fed overweight mice, RK preload reduced peak glucose levels in response to sucrose administration in males. RK did not reduce AUC compared with control. This could suggest that RK has insignificant *in vivo α*-glucosidase inhibitor actions rather than clinically significant *α*-glucosidase inhibitor actions (Xiong et al., 2018). One limitation is that we only examined the glucosidase inhibitory action at one dose and we observed an RK-induced increase in female mice. Notably, we did not observe alterations in gastric emptying or intestinal transit time with acute RK (200 mg/kg) in mice fed with a HFD for 2 weeks. The 2-week time was chosen for gastric emptying and intestinal transit time studies, because this time point would represent a hyperphagic phase of HFD-induced weight gain. Our findings, taken together, suggest that further *in vivo* studies regarding the altering the blood glucose levels effect with acute and chronic RK needs additional experimental attention. Moreover, the observed shift in blood glucose levels in female mice could be a sex-dependent effect that has adverse consequences when RK is used as a dietary supplement for weight loss in humans.

Adipose tissue is the main site for storing excess energy inputs and plays a critical role in the metabolic regulation and development of chronic inflammation by producing varieties of hormones and cytokines. Inflammation response in adipose tissue is also an important cause for insulin resistance (Hodson et al., 2008). Given that daily dosing of RK reduced adiposity, we hypothesized that RK may prevent inflammation in WAT and improve insulin sensitivity and may also affect adiposity metabolism-related adipokines gene expression. Studies on 3T3-L1 adipocytes have shown that RK inhibits adipogenesis and promotes lipolysis and fatty acid oxidation (Park, 2010, 2015; Leu et al., 2017). In this study, we examined the expression of *Lipe*, a hormone-sensitive lipase that is a major enzyme that catabolizes triglycerides during lipolysis (Frühbeck et al., 2014; Kim et al., 2016). However, there is only a trend increase of *Lipe* in mice dosed with RK. Apelin has been found widely expressed in many tissues, such as white adipose tissue (Kawamata et al., 2001). In WAT, apelin is detectable in preadipocytes; its expression is increased during adipogenesis and influenced by adipocyte size. Apelin is also upregulated by hyperinsulinemia and important for insulin sensitivity maintenance (Boucher et al., 2005; Yue et al., 2010). Studies using exogenous apelin treatment indicate that apelin negatively regulates lipolysis (Yue et al., 2011; Than et al., 2012; Bertrand et al., 2015). We have confirmed that orally administered RK reduces *apelin* expression in epididymal white adipose tissue in HFD-fed male mice (Mehanna et al., 2018). In addition, in our study, apelin expression was positively correlated with fat mass in RK-treated mice (r = 0.7228, *p* = 0.043). However, plasma insulin levels were highest in the vehicle-treated HFD-fed group but not significantly different from the RK-treated HFD-fed group. The reduction of apelin expression may indicate the anti-adipogenesis effect of RK (Park, 2015; Tsai et al., 2017). This may be, at least partially, attributed to the high bioavailability of RK and distribution to white adipose tissue (Zhao D. et al., 2020). However, it is necessary to investigate the physiological function of the endogenous apelin changes induced by RK to determine the direct relationship of apelin with whole body insulin resistance.

The metabolic signature of RK indicates an influence on peroxisome proliferator-activated receptor alpha (PPAR*α*) expression. PPAR*α* is a ligand activated transcription factor and highly expressed in the liver, brown adipose tissue, heart, and kidney (Braissant et al., 1996). Hepatic PPAR*α* is a major regulator for fatty acid oxidation, lipogenesis, and system inflammation (Pawlak et al., 2015), and increase in PPAR*α* in brown adipose tissue plays a role in lipogenesis and thermogenesis (Iizuka et al., 2013; Miranda et al., 2020). In our current study, RK treatment increased *Pparα* gene expression in the liver, and this may suggest a role of RK in hepatic lipid metabolism regulation (Wang et al., 2012). Raspberry ketone has been demonstrated to increase hepatic PPAR*α* and has a protective role in the development of nonalcoholic steatohepatitis (NASH) in rats (Wang et al., 2012). In our present study, it also worth noting that RK increases PPAR gamma coactivator 1, or PGC-1, in epididymal tissue when compared with HFD control. PGC-1interacts with PPAR*α* to coactivate enzyme genes that are involved in mitochondrial fatty acid oxidation (Lemberger et al., 1996; Vega et al., 2000). The effect of RK on PGC-1 expression suggests it might mediate RK promotion of fatty acid oxidation, a notion that requires further investigation (Park, 2010). A further study needs to investigate our finding that despite the putative action of RK on adipose lipid metabolism and no treatment difference in cumulative caloric intake, in this study RK has no effect on plasma concentrations of triglyceride, free fatty acid, or total cholesterol, which have been reported to represent either dietary intake or mobilization from adipose tissue (Durrington et al., 1977; Coppack et al., 1992; Hodson et al., 2008; Dimitriadis et al., 2016).

Dietary supplements marketed for weight loss are at risk for overuse, since individuals trying to achieve rapid weight loss could attempt to exceed the recommended dosage provided by the manufacturer. One cautionary limitation in the interpretation of our study is the dose-specific effects of RK. While conversion of animal doses to human doses is not entirely straightforward, using the human equivalent dose based on body surface is a rather well-accepted starting point for species extrapolation (Nair and Jacob, 2016). In this study, the mice were daily orally gavaged with 200 mg/kg of RK, which is based on body surface area equivalent to 16 mg/kg (or 1,040 mg per 65 kg) for humans (Nair and Jacob, 2016). As such, these report effects on RK are dose-specific at 200 mg/kg in male mice, since with acute oral dosing at a half-log increment of 640 mg/kg we previously observed ∼40% mortality, adipose atrophy, splenic abnormalities, and thymus involution (Hao et al., 2020).

## Data Availability Statement

The raw data supporting the conclusions of this article will be made available by the authors, without undue reservation.

## Ethics Statement

The animal study was reviewed and approved by Institutional Animal Care and Use Committee of Rutgers University (OLAW #A3262-01); Protocol #999900014.

## Author Contributions

NB and LH designed the study. LH performed the experiments, analyzed the data, and drafted the manuscript. NB analyzed the data and drafted and edited the manuscript. Both authors contributed to the article and approved the submitted version.

## Conflict of Interest

The authors declare that the research was conducted in the absence of any commercial or financial relationships that could be construed as a potential conflict of interest.

## Publisher’s Note

All claims expressed in this article are solely those of the authors and do not necessarily represent those of their affiliated organizations, or those of the publisher, the editors and the reviewers. Any product that may be evaluated in this article, or claim that may be made by its manufacturer, is not guaranteed or endorsed by the publisher.

## Abbreviations

Adra1a: adrenoceptor alpha-1A
Adra1b: adrenoceptor alpha-1B
Adra2b: adrenoceptor alpha-2B
Adra2c: adrenoceptor alpha-2C
Alox15: arachidonate 15-lipoxygenase
Apln: apelin
Aplnr: apelin receptor
b3AR: beta-3 adrenergic receptor
Gabrg2: gamma-aminobutyric acid receptor subunit gamma-2
Gria1: glutamate ionotropic receptor AMPA-type subunit 1
Hmox1: heme oxygenase
ITT: insulin tolerance test
Il-6: interleukin 6
Lipe: hormone sensitive lipase
Lpl: lipoprotein lipase
Npy: neuropeptide Y
OGTT: oral glucose tolerance test
Pomc: proopiomelanocortin
Ppara: peroxisome proliferator activated receptor alpha
Pparg: peroxisome proliferator activated receptor gamma
Pparg1ca: peroxisome proliferator-activated receptor-gamma coactivator (PGC)-1alpha
RK: raspberry ketone
TNF: tumor necrosis factor
Ucp1: uncoupled protein 1
Ucp2: uncoupled protein 2
Veh: vehicle.
