# Supplementary material for: Reduced Body Fat and Epididymal Adipose Apelin Expression Associated With Raspberry Ketone [4-(4-Hydroxyphenyl)-2-Butanone] Weight Gain Prevention in High-Fat-Diet Fed Mice
Source: Front Physiol. 2021 Nov 23;12:771816. doi: 10.3389/fphys.2021.771816 (PMC8650585; doi:10.3389/fphys.2021.771816)
Supplement: Supplementary file 1 [file Data_Sheet_1.docx]

Supplementary Figure: Experimental design for 12‐week treatment with raspberry ketone in C57BL6 mice.

| Supplementary Table. Completed blood cell counts for mice with raspberry ketone or vehicle daily dosing for a period of 12 weeks | | | | |
| --- | --- | --- | --- | --- |
|  | LFD_Veh | HFD_Veh | HFD_RKw4 | HFD_RK |
| \| WBC,10^3^/μL \| \| --- \| \| NEU, 10^3^/μL \| \| LYM, 10^3^/μL \| \| MONO, 10^3^/μL \| \| EOS, 10^3^/μL \| \| BAS, 10^3^/μL \| \| NEU % \| \| LYM % \| \| MONO % \| \| EOS % \| \| BAS % \| \| RBC, 10^6^/μL \| \| HGB, g/dL \| \| HCT% \| \| MCV, fL \| \| MCH, pg \| \| MCHC, g/dL \| \| RDW % \| \| PLT, 10^3^/μL \| \| MPV, fL \| | \| 1.07 ± 0.16 \| \| --- \| \| 0.38 ± 0.06 \| \| 0.55 ± 0.08 \| \| 0.066 ± 0.01 \| \| 0.04 ± 0.01 \| \| 0.033 ± 0.01 \| \| 34.99 ± 1.24 \| \| 50.94 ± 2.24 \| \| 6.83 ± 1.29 \| \| 4.01 ± 0.82 \| \| 3.23 ± 0.57 \| \| 9.39 ± 0.24 \| \| 15.44 ± 0.34 \| \| 46.31 ± 1.02 \| \| 49.34 ± 0.31 \| \| 16.44 ± 0.13 \| \| 33.33 ± 0.20 \| \| 14.19 ± 0.30 \| \| 1004.86 ± 58.52 \| \| 5.2 ± 0.07 \| | \| 0.99 ± 0.15 \| \| --- \| \| 0.29 ± 0.08 \| \| 0.57 ± 0.06 \| \| 0.03 ± 0.01 \| \| 0.051 ± 0.02 \| \| 0.04 ± 0.02 \| \| 26.39 ± 3.25 \| \| 62.73 ± 5.33 \| \| 3.49 ± 0.79 \| \| 4.70 ± 1.33 \| \| 2.70 ± 1.49 \| \| 9.19 ± 0.65 \| \| 15.28 ± 0.91 \| \| 44.53 ± 3.12 \| \| 48.48 ± 0.36 \| \| 16.80 ± 0.35 \| \| 34.64 ± 0.65 \| \| 14.10 ± 0.15 \| \| 763.63 ±108.78 \| \| 5.18 ± 0.11 \| | \| 0.79 ± 0.09 \| \| --- \| \| 0.37 ± 0.09 \| \| 0.36 ± 0.04* \| \| 0.04 ± 0.01 \| \| 0.01 ± 0.01 \| \| 0.02 ± 0.01 \| \| 42.70 ± 5.33& \| \| 47.11 ± 5.39 \| \| 4.60 ± 0.63 \| \| 2.26 ± 0.49 \| \| 3.33 ± 0.76 \| \| 9.73 ± 0.15 \| \| 15.53 ± 0.18 \| \| 46.88 ± 0.69 \| \| 48.19 ± 0.31 \| \| 15.97 ± 0.13 \| \| 33.14 ± 0.27 \| \| 14.25 ± 0.08 \| \| 1040.88 ± 29.45 \| \| 4.99 ± 0.07 \| | \| 0.72 ± 0.16 \| \| --- \| \| 0.29 ± 0.07 \| \| 0.33 ± 0.06*& \| \| 0.03 ± 0.01 \| \| 0.03 ± 0.02 \| \| 0.05 ± 0.02 \| \| 37.29 ± 3.43 \| \| 49.14 ± 4.21 \| \| 4.60 ± 0.93 \| \| 3.85 ± 0.96 \| \| 5.13 ± 1.40 \| \| 8.73 ± 0.40 \| \| 14.16 ± 0.61 \| \| 42.55 ± 1.86 \| \| 48.79 ± 0.30 \| \| 16.24 ± 0.08 \| \| 33.30 ± 0.20 \| \| 13.65 ± 0.13 \| \| 841.00 ±120.83 \| \| 5.20 ± 0.09 \| |
| raspberry ketone (RK); vehicle (Veh); high fat diet (HFD); low fat diet (LFD); white blood cell count (WBC); neutrophils (NEU); lymphocytes (LYM); monocytes (Mono); eosinophils (EOS); red blood cell count (RBC); hemoglobin concentration (HGB); hematocrit (HCT); mean corpuscular volume (MCV); mean corpuscular hemoglobin (MCH); mean corpuscular hemoglobin concentration (MCHC); red blood cell count distribution width coefficient of variation (RDW); platelet count (PLT); mean platelet volume (MPV). Data are represented as means ± SEM. *p<0.05 vs LFD_Veh, & p < 0.05 vs. HFD_Veh; One way ANOVA followed by Newman Keuls post hoc testing. | | | | |
